# Descending interneurons of the stick insect connecting brain neuropiles with the prothoracic ganglion

**DOI:** 10.1371/journal.pone.0290359

**Published:** 2023-08-31

**Authors:** Jens Goldammer, Ansgar Büschges, Volker Dürr

**Affiliations:** 1 Department of Animal Physiology and Neurobiology, Institute of Zoology, Biocenter Cologne, University of Cologne, Cologne, Germany; 2 Department of Biological Cybernetics, Bielefeld University, Bielefeld, Germany; Georgia State University, UNITED STATES

## Abstract

Stick insects respond to visual or tactile stimuli with whole-body turning or directed reach-to-grasp movements. Such sensory-induced turning and reaching behaviour requires interneurons to convey information from sensory neuropils of the head ganglia to motor neuropils of the thoracic ganglia. To date, descending interneurons are largely unknown in stick insects. In particular, it is unclear whether the special role of the front legs in sensory-induced turning and reaching has a neuroanatomical correlate in terms of descending interneuron numbers. Here, we describe the population of descending interneurons with somata in the brain or gnathal ganglion in the stick insect *Carausius morosus*, providing a first map of soma cluster counts and locations. By comparison of interneuron populations with projections to the pro- and mesothoracic ganglia, we then estimate the fraction of descending interneurons that terminate in the prothoracic ganglion. With regard to short-latency, touch-mediated reach-to-grasp movements, we also locate likely sites of synaptic interactions between antennal proprioceptive afferents to the deutocerebrum and gnathal ganglion with descending or ascending interneuron fibres. To this end, we combine fluorescent dye stainings of thoracic connectives with stainings of antennal hair field sensilla. Backfills of neck connectives revealed up to 410 descending interneuron somata (brain: 205 in 19 clusters; gnathal ganglion: 205). In comparison, backfills of the prothorax-mesothorax connectives stained only up to 173 somata (brain: 83 in 16 clusters; gnathal ganglion: 90), suggesting that up to 60% of all descending interneurons may terminate in the prothoracic ganglion (estimated upper bound). Double stainings of connectives and antennal hair field sensilla revealed that ascending or descending fibres arborise in close proximity of afferent terminals in the deutocerebrum and in the middle part of the gnathal ganglia. We conclude that two cephalothoracic pathways may convey cues about antennal movement and pointing direction to thoracic motor centres via two synapses only.

## Introduction

Stick insects readily orient towards visual cues [1–4], show visually induced turning [5, 6] and exhibit tactually induced climbing behaviour, including aimed front leg movements [7, 8]. All of these sensory-induced changes in walking direction and spatial coordination must involve descending neural pathways that connect sensory processing centres of the brain with motor networks of the thoracic ganglia [9, 10].

The stick insect *Carausius morosus* (de Sinéty, 1901) [11] is an established study organism in insect motor physiology (e.g. [12–14]). However, in contrast to the detailed knowledge of its thoracic reflex pathways and pre-motor networks (for review see [15]) and flexibility of motor behaviour (for review see [16, 17]) very little is known about the neural pathways from the head ganglia to the ventral nerve cord. In some insect orders, the population of descending interneurons (DNs) of the brain has been charted on the light microscopy level to some extent (locusts: [18]; crickets: [19]; cockroaches: [20, 21]; blowfly: [22]; fruit fly: [23, 24]). Recently, an entire DN population has been analysed in a connectome dataset of a *Drosophila* male adult ventral nerve chord [25].

In contrast, no DNs of the stick insect brain and only a small number of DNs of the gnathal ganglion [26] have been described so far. In order to amend this, the first objective of the present study is to provide an anatomical overview of the number, soma locations, and major arborisation regions of DNs in the head ganglia of the stick insect, i.e., the cerebral ganglion (CRG; typically called the brain in hemimetabolous insects) and the gnathal ganglion (GNG; synonymous to suboesophageal ganglion; [27]).

With regard to sensory-induced motor behaviour in stick insects, a number of studies have emphasised a special role of the front legs. Examples include an increased likelihood of short steps [28], weaker coupling to the rear legs in temporal and spatial inter-leg coordination [29, 30]. pronounced searching-movements (e.g. [31, 32]), an initiating role in visually-induced turning [4, 5] and tactually-induced reach-to-grasp movements towards tactile contact locations [7]. Together, this data suggests an important function of front legs in near-range exploration (for review, see [16]), including the integration of descending sensory cues for goal-directed adaptation of locomotion. A particularly compelling example of this descending sensory integration is the short-latency re-targeting of ongoing swing movements in front legs: within 40 ms of an antennal contact event, the ipsilateral front leg may adjust the direction of its foot trajectory towards the contacted location [7], effectively turning a walking step into an aimed reach-to-grasp movement. In order to see if descending sensory control of front leg movements is mirrored by neuroanatomical properties of descending neural pathways, the second objective of this paper is to estimate the fraction of DNs that terminates in the prothoracic ganglion.

As nocturnal animals, stick insects rely heavily on antennal tactile exploration for orientation. It is therefore important to know how DNs are connected with antennal sensory processing centres in the brain like the dorsal lobe (DL) and the ventral area of flagellar afferents (VFA), neuropils for integrating antennal hair field and flagellar signals [33]. Accordingly, we will give special emphasis to the neural pathways conveying information about antennal posture and movement. Ache and Dürr [34] have proposed an electrophysiological classification of DNs that encode antennal joint angle and/or angular velocity. A least some of the DNs recorded by them had sensory-induced spike latencies less than 20 ms in the prothoracic ganglion, indicating the possibility of a direct transfer of proprioceptive afferent information to DNs. Indeed, two individually identified DNs in the GNG of the stick insect which encode antennal movement velocity, the contralateral ON-type velocity-sensitive DN (cONv) and ipsilateral ON-type velocity-sensitive DN (iONv), both of which were shown to have dendritic branches in close proximity to afferent terminals of antennal hair field sensilla [26]. Given the behavioural relevance of the antennal tactile sense in stick insects, and the particular evidence suggesting short-latency encoding of antennal proprioceptive cues for tactually induced reach-to-grasp movements, our third objective is to identify all neuropil regions within the CRG and/or GNG where direct synaptic contacts between antennal proprioceptor afferents and DNs may occur. We will focus on antennal hair field afferents because they are known to encode antennal pointing direction and movement velocity in cockroaches [35], strongly affect antennal movement in stick insects [36] and at least one DN of the gnathal ganglion (cONv) shows antennal-movement-related activity that is partly driven by afferent activity of proprioceptive hair fields [37].

In order to address the first two objectives, we stained neck connectives and prothorax-mesothorax connectives of *C*. *morosus* with fluorescent tracers and determined the number, distribution and major arborisation regions of DNs in the CRG and GNG. By relating the DN numbers stained by the two types of connectives, we provide an upper bound estimate of the fraction of DNs that terminates in the prothoracic ganglion. With regard to the third objective, we combined stains of connectives with backfills of antennal hair field afferents. As a first step, this allowed us to identify a small number of regions in the deutocerebrum and in the middle part of the gnathal ganglion where synaptic interactions between hair field afferents and DNs and ANs could occur, as judged from confocal microscopy.

## Materials and methods

### Animals

Experiments were performed on adult female stick insects, *Carausius morosus* [11], taken from colonies maintained at the Universities of Cologne and Bielefeld. The colonies were kept at temperatures between 20°C and 25°C in an artificial light/dark cycle, and fed *ad libitum* with blackberry leaves (*Rubus fructiosus*).

### Staining of hair field afferents and descending interneurons

Hair field afferents (HF) were stained as described in detail in [33]. The stick insect carries seven antennal hair fields, three on the scape and four on the pedicel (for terminology see [36]). Out of these seven HFs, we performed only stainings of scapal hair plate dorsal (sHPd) and scapal hair plate ventral (sHPv) afferents.

For labelling of descending interneurons (DN) of the cerebral ganglion (CRG; synonymous to supraoesophageal ganglion) and gnathal ganglion (GNG; synonymous to suboesophageal ganglion; [27]), the animal was fixed ventral side up with plasticine. A small window was cut into the sternum and postmentum between the GNG and prothoracic ganglion or into the sternal plates between the pro- and mesothoracic ganglia to expose the connectives of the ventral nerve cord. One connective was cut and surrounded by a well, made of Vaseline petroleum jelly. At first, the well was filled with distilled water. After 5 min., water was replaced by a tetramethylrhodamine dextran solution (TRDA; Invitrogen, Eugene, OR; 5% in distilled water). In some preparations, scapal HPs were stained in addition with a fluorescein dextran solution (FDA; Invitrogen, Eugene, OR; 10% in distilled water). Animals were kept for up to 96 hours at 4–7°C before the CRG and GNG were removed, and cleaned from fat and connective tissue. Ganglia were then exposed to 20% Triton X-100 (Fluka, Buchs, Switzerland) in a fixative for 20 minutes (4% paraformaldehyde or 4% Roti-Histofix; Roth, Karlsruhe, Germany) and subsequently placed in pure fixative for 2 hours at room temperature [38]. Afterwards, ganglia were washed (3 x 10 minutes in 0.1 M phosphate-buffered saline, PBS, pH 7.2), dehydrated in an ascending ethanol series (30%, 50%, 70%, 90%, 2 x 100%, 10 min each step), mounted on microscopic slides with cavity (Thermo Scientific, Braunschweig, Germany), and cleared with methyl salicylate (Roth, Karlsruhe, Germany). The prothoracic ganglion was also removed if a connective between the pro- and mesothoracic ganglia was stained.

### Anti-synapsin immunohistochemistry

Several CRG whole-mounts were incubated with an antibody directed against the Drosophila synaptic protein synapsin [39]. To facilitate the antibody penetration, the ganglion sheath was treated for 30 s with small crystals of a proteolytic enzyme (Pronase E, Merck, Darmstadt, Germany). The enzyme was washed out by repeated rinsing with stick insect saline. Then, the detached sheath was manually removed with fine forceps and a pair of sharp scissors. Specimens were then fixed and washed as described above. Afterwards, ganglia were preincubated in 0.1 M PBS containing 5% normal goat serum (S1000, Linaris, Wertheim, Germany), 1% Triton-X 100 (Fluka, Buchs, Switzerland) and 0.1% sodium azide (AppliChem) for 1–2 hours to block unspecific antibody binding. The same solution was used for washes, and the dilution of primary and secondary antibodies. The monoclonal mouse anti-synapsin antibody SYNORF1 (kindly provided by Erich Buchner, University of Würzburg, Germany) was applied at a dilution of 1:25 for 2 x 3 days at 4°C. Subsequently, three washes of 2 hours each were carried out and ganglia were incubated with DyLight 633-conjugated goat anti-mouse IgG antibody (ThermoScientific) diluted 1:100 in blocking-solution for 2.5 days at 4°C. Ganglia were washed 3 x 1 hour in PBS, dehydrated and cleared as described above.

### Image acquisition

Image stacks of whole-mounts were captured with a confocal laser scanning microscope (LSM 510; Carl Zeiss, Germany; Imaging Facility Biocenter Cologne) equipped with Plan-Neofluar 10x (0.65 NA) and Plan-Apochromat 20x (0.75 NA) objectives. FDA, TRDA, and DyLight 633 were imaged with 488, 543, and 633 nm excitation, respectively. Emission of TRDA was collected through a 560 long-pass (LP) filter or a 560–630 nm band-pass (BP) filter for double or triple labeled ganglia with DyLight 633 and FDA. For emission of FDA and DyLight 633 a 505–530 BP and a 650 LP filter was used, respectively. For documentation, optical sections were scanned in intervals between 1 and 5 μm with the Zeiss LSM 510 microscope. In several CRG and GNG images, the ganglion outline was captured by scanning the autofluorescence of the tissue basic with 488 nm excitation. Projections and depth colour-coded images were generated with the Zeiss LSM Image Browser (v4.2.0.121).

### Terminology of fibre tracts, neural tissue, and descending interneurons

The terminology for sensory fibre tracts in the brain of the stick insect was adopted from the honeybee nomenclature proposed by Suzuki [40] and is described in detail in [33]. Stick insects possess prognathous mouth parts, which is why the cerebral ganglion (CRG) is tilted backwards in the adult head capsule. As a consequence, the neuraxis (n-) is pointing rearward, and n-ventral CRG neuropils are facing dorsally inside the head. Throughout this paper, all central projections stained in the CRG as well as general neuroanatomical descriptions of locations in the CRG follow the conventions proposed by Ito et al. [27]. Note that this differs from the original description of the stick insect brain nerves by Dürr et al. [41], where anatomical locations coincide with their *in situ* location. Neurons with a cell body in the CRG and a descending axon will be named Descending Interneurons (DNs) throughout this study. Other than the terms Descending Brain Neuron [19] or Descending Neuron [20] the term DN applies only to interneurons, but does not exclude neurons with cell bodies in the GNG that have a descending axon [18]. As in all experiments described here, only one connective was stained per specimen, soma clusters are named ipsilateral (i) or contralateral (c) with respect to the dyed connective. Where possible, cell body clusters of DNs within the CRG were named according to the nomenclature of Staudacher [19].

## Results

### DNs projecting to the prothoracic ganglion

**Fig 1:**
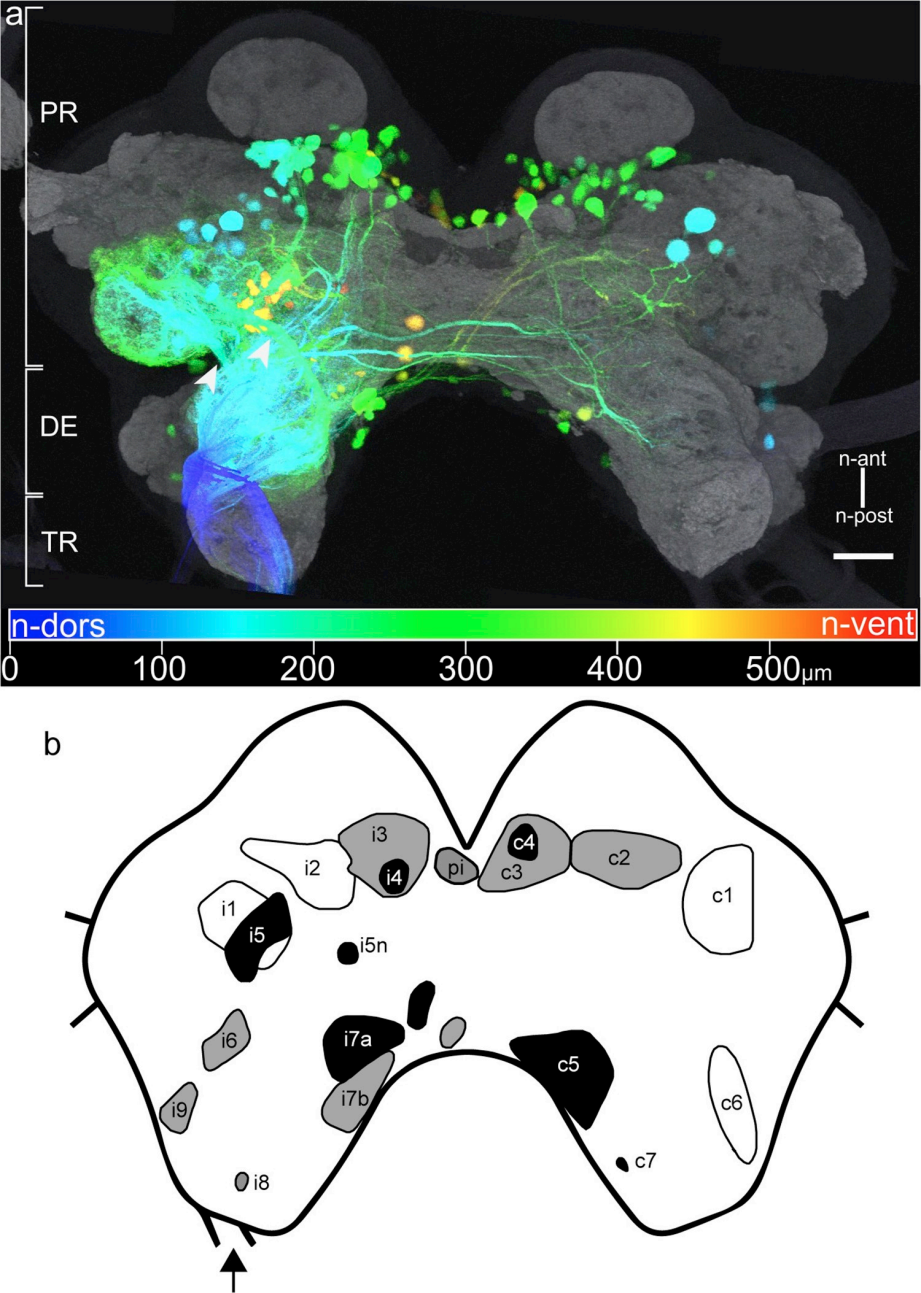
Descending interneurons (DN) in the CRG after staining a neck connective. Stick insects possess prognathous mouth parts, which is why the cerebral ganglion (CRG) is tilted backwards in the adult head capsule. As a consequence, the neuraxis (n-) is pointing rearward, and n-ventral CRG neuropils are facing dorsally inside the head. a: Depth colour-coded whole-mount of the CRG, combined with anti-synapsin immunostaining (gray). The CRG outline was captured by scanning the autofluorescence of the tissue (dark gray). Depth is encoded from n-dorsal to n-ventral as blue to red. Brackets indicate approximate borders of the tritocerebrum (TR), deutocerebrum (DE), and protocerebrum (PR). Arrowheads mark two thick fibre bundles of potential axons of ascending interneurons (AN) or primary sensory neurons projecting from the gnathal ganglion and the ventral nerve cord into the lateral PR. b: Schematic drawing of the soma cluster distribution in the CRG, ipsi- (i) and contralaterally (c) to the side of the dyed connective (arrow). pi: pars intercerebralis cluster. Shades of gray indicate n-dorsoventral location of the clusters: n-dorsal = white with black outline; intermediate = gray; n-ventral = black. The two unlabelled clusters in the n-posterior mid region of the PR were found rarely. Scale bar = 100 μm.

**Fig 2:**
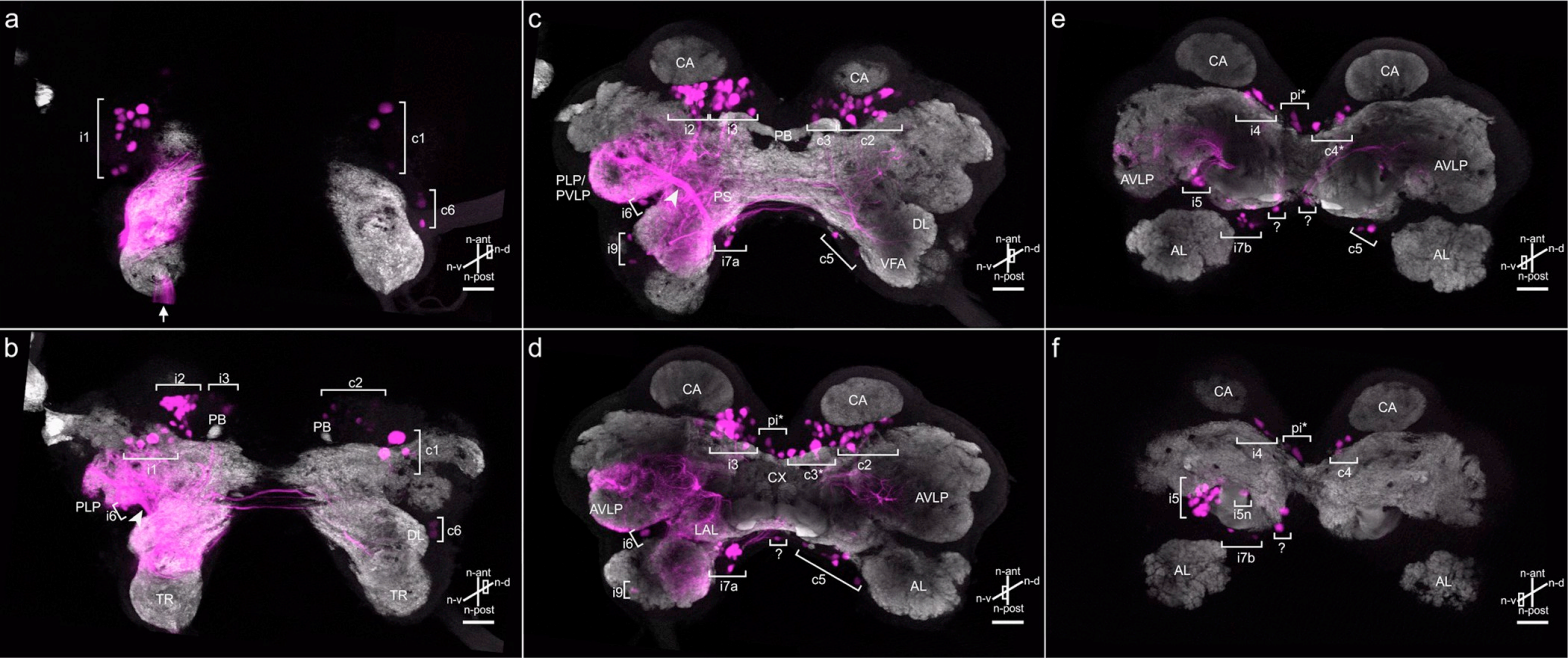
Thick optical CRG sections with DNs stained through a neck connective. Connective backfill (magenta) combined with anti-synapsin immunostaining (gray). a-f: Optical sections (same whole-mount shown in Fig 1A) from n-dorsal (a) to n-ventral CRG parts (f). For an explanation of the term neuraxis (n-) please see Fig 1. Arrow in (a) marks the site of the dyed neck connective. Labels indicate soma clusters and prominent CRG neuropils. AL: antennal lobe; CA: calyces; CX: central complex; DL: dorsal lobe; PB: protocerebral bridge; VFA: ventral area of flagellar afferents. Arborisations of DNs occur in the posterior lateral protocerebrum (PLP), the posterior ventrolateral protocerebrum (PVLP), the anterior ventrolateral protocerebrum (AVLP), the posterior slope (PS), the lateral accessory lobe (LAL), the ipsi- and contralateral DLs and VFAs, and in the medial contralateral protocerebrum (b-e). Arrowheads in (b) and (c) mark two thick fibre bundles of potential axons of ascending interneurons (AN) or primary sensory neurons projecting from the gnathal ganglion and the ventral nerve cord into the lateral PR. Asterisks mark clusters with undefined borders. Question marks indicate solitary neurons. The dark CRG outline, e.g. the cell body rind, was captured by scanning the autofluorescence of the tissue. Scale bars = 100 μm.

Backfilling the neck connective stained 164 to 205 (N = 5; median: 176) somata in the CRG. Fig 1 and S1 Fig show two depth colour-coded CRG specimens with labelled DNs, whereas in Fig 2 thick optical brain sections are shown. DN numbers and their clusters are summarized in Table 1. In the brain, these neurons are located in 19 soma clusters, eleven ipsilaterally (i1-9), the pars intercerebralis cluster (pi) and seven contralateral clusters (c1-7). They are distributed in n-dorsal, intermediate and n-ventral regions of the trito- (TR), deuto- (DE), and protocerebrum (PR). Many stained cell bodies are located near the ipsi- and contralateral mushroom body calyces. The soma clusters i9, i5n and c7 may not be clusters in a strict sense because they often contain only one cell body. Nevertheless, owing to their occurrence in similar regions in different specimens, and the existence of similar clusters in crickets and cockroaches, they were defined as clusters. In general, the cluster borders may not be discernible easily, mostly due to a smooth transition in depth (see depth colour-coded image). Therefore, the cell body counts for i2-i4, i7a-i7b, and c2-c4 have to be considered with care (Table 1). The cell body diameters range between 10 μm to at least 50 μm. One of the largest somata was found contralaterally in cluster c1. It possesses the largest cell body fibre, measuring ~10 μm in diameter.

**Table 1:** Soma clusters with numbers of CRG DNs and their location, after staining a neck connective for five specimens (N = 5).

	**i1**	**i2** ^**1**^	**i3** ^**1**^	**i4** ^**1**^	**i5**	**i5n**	**i6**	**i7a** ^**1**^	**i7b** ^**1**^	**i8**	**i9**
	**dPR**	**dPR**	**intPR**	**vPR**	**vPR**	**vPR**	**v/intDE/PR**	**vDE**	**intDE**	**int/dTR**	**intDE**
**1**	8	21	20	5	25	2	3	7	6	1	3
**2**	9	25	22	7	22	2	3	9	7	1	0
**3**	11	22	38	6	28	0	4	10	10	1	2
**4**	15	19	29	3	19	1	2	8	9	1	2
**5**	11	21	23	4	24	1	3	8	3	1	4
***max*.**	*15*	*25*	*38*	*7*	*28*	*2*	*4*	*10*	*10*	*1*	*4*
	**pi** ^**1**^	**c1**	**c2** ^**1**^	**c3** ^**1**^	**c4** ^**1**^	**c5**	**c6**	**c7**	**single cells**	**total #**	
	**vPR**	**dPR**	**d/intPR**	**intPR**	**vPR**	**vDE**	**dDE/TR**	**vDE**	**various**		
**1**	4	11	15	12	9	7	6	1	2	**168**	
**2**	5	7	22	9	1	7	6	2	2	**168**	
**3**	6	13	17	18	6	6	6	1	0	**205**	
**4**	6	5	11	24	8	7	4	2	2	**177**	
**5**	5	6	13	23	2	4	3	0	5	**164**	
***max*.**	*6*	*13*	*22*	*24*	*9*	*7*	*6*	*2*	*5*	***238***	

d = n-dorsal; int = intermediate; v = n-ventral; DE = deutocerebrum; PR = protocerebrum; TR = tritocerebrum.

^1^Note that cluster borders are difficult to delimit in practice. Few cell bodies counted for one cluster may have had to be assigned to the neighbouring cluster.

The distribution and branching pattern of DNs can be observed clearly in the anti-synapsin immunostained images (Fig 2). Note that, due to the huge amount of heavily stained branches, it is not possible to distinguish between DN branches, terminal fibres of ascending interneurons (AN), and terminal fibres of primary sensory neurons. Nevertheless, a considerable amount of branching can be observed ipsilaterally in the posterior lateral protocerebrum (PLP), in the posterior ventrolateral protocerebrum (PVLP) and in the anterior ventrolateral protocerebrum (AVLP) (Fig 2B–2E). Two thick bundles of branches are particularly apparent, potentially belonging to ANs, that travel ipsilaterally to arborise and terminate in the lateral PR (arrowheads in Figs 1A and 2B, 2C). Few weakly labelled branches run in the direction of the ipsilateral optic lobe (not shown). Numerous dendritic branches were also observed in the ipsi- and contralateral dorsal lobes (DLs) and the ventral area of flagellar afferents (VFA) in the DE (Fig 2B and 2C). Extensive arborisations were stained in a region between the central complex (CX) and the ipsilateral antennal lobe (AL; Fig 2D), known as the lateral accessory lobe in moths, locusts, and *Drosophila* (LAL; [42, 43]). Ipsi- and contralaterally to the filled connective, dendritic branches were found in the intermediate and medial PR (Fig 2C–2E). No dendritic arborisations were observed in the AL, the CX and the mushroom bodies, indicating that none of these neuropils provide direct information to descending interneurons.

**Fig 3:**
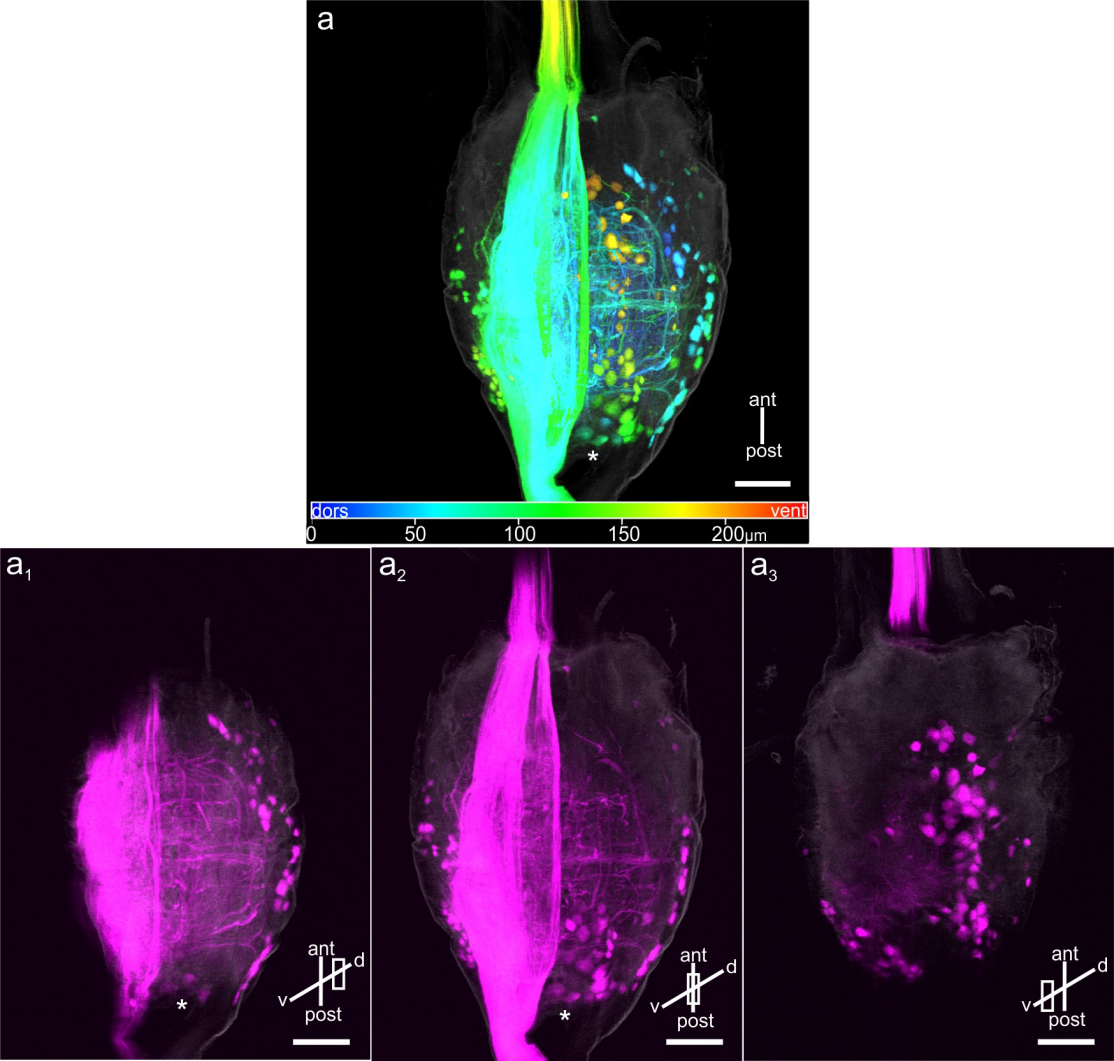
GNG whole-mount, showing DNs stained through a neck connective. a: Depth-colour coded specimen. Depth is encoded from dorsal to ventral as blue to red. Outline (gray) was captured by scanning the autofluorescence (also in a1-a3). About 200 cell bodies were dyed ipsi- and contralaterally to the stained connective and along the GNG midline. Asterisk marks the position of DUM cell bodies. a1-a3: Thick optical GNG sections of specimen shown in (a) from dorsal (a1) to ventral regions (a3). Contralateral cell bodies are located mainly dorsolaterally, but also in intermediate and ventral regions (a1-a3). Ipsilateral cell bodies were stained posterolaterally in intermediate and ventral parts (Fig 3A2–3A3). Along the GNG midline, the majority of stained cell bodies are located ventrally in the cell body rind (a2-a3). Dense arborisations of branches occur in the core GNG neuropil (a1-a2). Asterisks mark the position of stained DUM cell bodies. Scale bars = 100 μm.

In the GNG, at least 161 and up to 205 (N = 5; median: 183) cell bodies were stained through a neck connective (Fig 3A). The cell bodies were mainly stained in three prominent regions at the GNG midline and ipsi- and contralaterally to the stained connective in lateral GNG parts, breaching the entire depth of the GNG (Fig 3A–3A3). At the posterior crotch of the GNG, several DUM neurons were stained. Their exact numbers could not be determined, but Stolz et al. [44] estimated about five to seven DUM neurons in the GNG of stick insects. Just after entering the GNG, fibres of descending and ascending neurons are running through different longitudinal tracts. Several fibres are crossing over to the contralateral side, forming different commissures. Extensive arborisations were found in the core neuropil of the GNG (Fig 3A1 and 3A2). In summary, a maximum of 410 pairs of DNs in the stick insect CRG and GNG may convey sensory signals from the head to thoracic motor centres.

### DNs projecting to the mesothoracic ganglion

**Fig 4:**
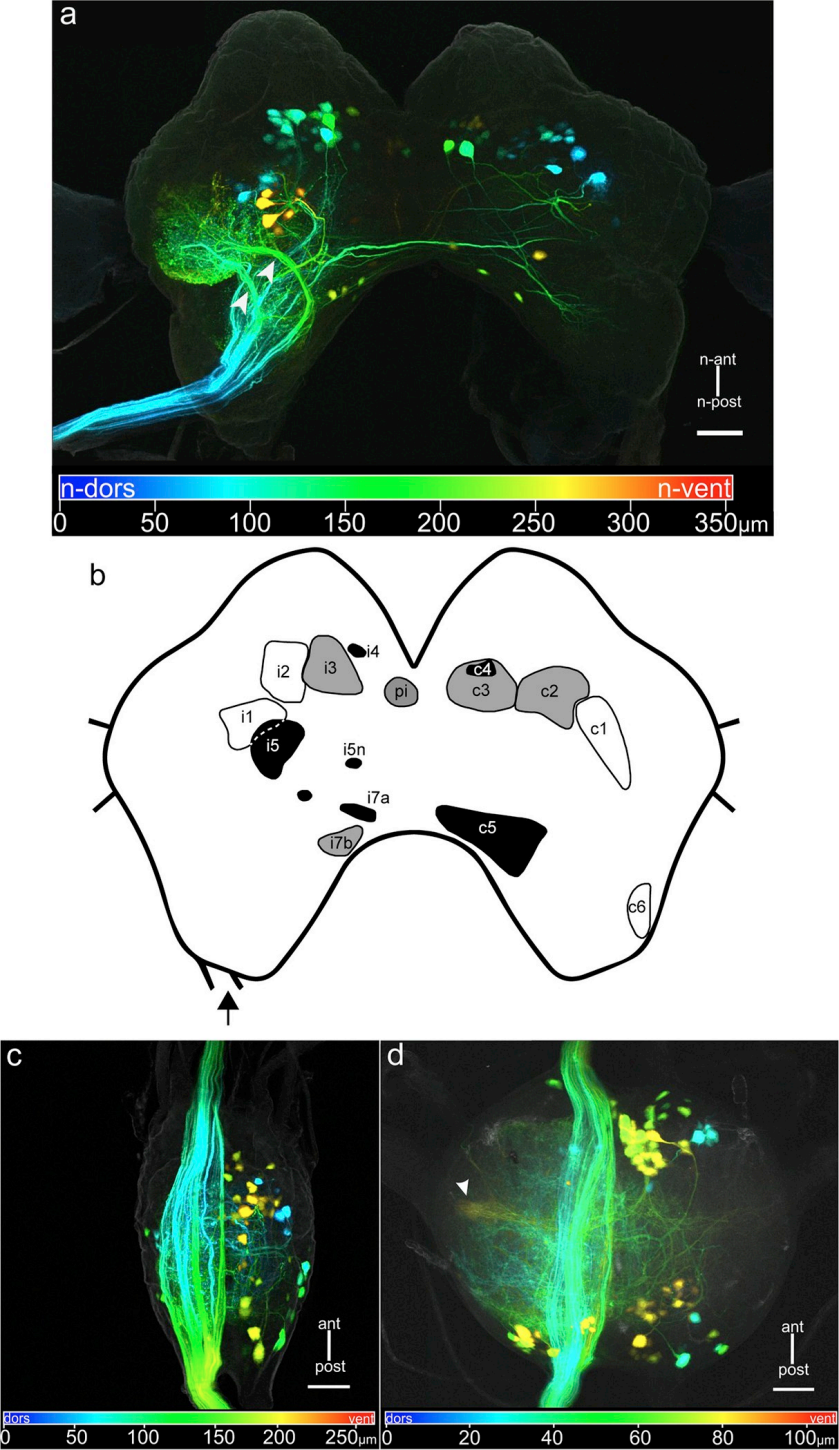
DNs in the CRG and GNG after staining the prothorax-mesothorax connective. a: Depth colour-coded CRG with minor distortion along z-axis. The CRG outline was captured by scanning the autofluorescence of the tissue (gray). Depth is encoded from n-dorsal to n-ventral as blue to red. Arrowheads mark two thick fibre bundles of potential ascending interneuron (AN) axons projecting from the gnathal ganglion and the ventral nerve cord into the lateral PR. b: Schematic drawing of the soma cluster distribution in the CRG, ipsi- (i) and contralaterally (c) to the side of the dyed connective (arrow). pi: pars intercerebralis cluster. Gray scale indicates the n-dorso-ventral location of the clusters; n-dorsal: white with black outline; intermediate: gray; n-ventral: black. For an explanation of the term neuraxis (n-) please see Fig 1. c: DNs in the GNG. The outline (gray) was captured by scanning autofluorescence. Depth coding as in (a). Approximately 80 cell bodies were dyed in different regions and clusters, the majority in ventral parts of the ganglion. d: Intersegmental interneurons in the prothoracic ganglion. Outline and depth coding as in (a) and (c). Approximately 100 cell bodies with axons to the mesothoracic ganglion were stained in ipsi- and contralateral ganglion regions. Arrowhead marks branches in the VAC, a prominent sensory neuropil. Scale bars = 100 μm.

Backfills of a prothorax- mesothorax connective revealed 51 to 83 DNs (N = 4; median: 68) in the CRG (Fig 4 and Table 2). They are distributed ipsilaterally in soma clusters i1-i7b, in the pars intercerebralis (pi) cluster, and contralateral clusters c1-c6. No cell bodies were observed in clusters i8, i9, and c7 (Table 2). Much like in staining of the neck connectives, arborisations of DNs were found in the ipsilateral PLP, PVLP, AVLP, the ipsilateral LAL, the DL and VFA neuropil on both brain hemispheres, and within the contralateral medial PR.

**Table 2:** Soma clusters with numbers of CRG DNs and their location, after staining a connective between pro- and mesothoracic ganglia for four specimens (N = 4).

	**i1**	**i2** ^**1**^	**i3** ^**1**^	**i4** ^**1**^	**i5**	**i5n**	**i6**	**i7a** ^**1**^	**i7b** ^**1**^	**i8**	**i9**
	**dPR**	**dPR**	**intPR**	**vPR**	**vPR**	**vPR**	**v/intDE/PR**	**vDE**	**intDE**	**int/dTR**	**intDE**
**1**	4	12	13	1	9	1	1	5	2	0	0
**2**	6	12	12	1	8	1	0	5	1	0	0
**3**	7	8	12	1	6	0	0	4	0	0	0
**4**	3	8	5	1	8	0	0	0	0	0	0
***max*.**	*7*	*12*	*13*	*1*	*9*	*1*	*1*	*5*	*2*	*0*	*0*
	**pi** ^**1**^	**c1**	**c2** ^**1**^	**c3** ^**1**^	**c4** ^**1**^	**c5**	**c6**	**c7**	**total #**		
	**vPR**	**dPR**	**d/intPR**	**intPR**	**vPR**	**vDE**	**dDE/TR**	**vDE**				
**1**	4	5	12	6	4	4	0	0	**83**		
**2**	2	2	7	5	1	4	0	0	**67**		
**3**	1	2	16	8	0	4	2	0	**71**		
**4**	4	3	9	4	2	4	0	0	**51**		
***max*.**	*4*	*5*	*16*	*8*	*4*	*4*	*2*	*0*	***94***		

d = n-dorsal; int = intermediate; v = n-ventral; DE = deutocerebrum; PR = protocerebrum; TR = tritocerebrum.

^1^Note that cluster borders are difficult to delimit in practice. Few cell bodies counted for one cluster may have had to be assigned to the neighbouring cluster.

In the GNG, at least 69 and up to 90 somata (median: 82; N = 4) were observed, with similar distributions and fewer arborisations in the neuropil than described above (Fig 4C). For matters of completeness, intersegmental neurons of the prothoracic ganglion were counted, too. The stainings showed 90 and up to 115 neurons (N = 4, median: 105), mainly ventrally and contralaterally to the filled connective (Fig 4D). Several neurons possess arborisations in the ipsilateral ventral association centre (VAC) of the prothoracic ganglion, a prominent sensory neuropil (Fig 4D arrowhead).

### Central projections of hair field fibres appear to be in close vicinity to branches of DNs

**Fig 5:**
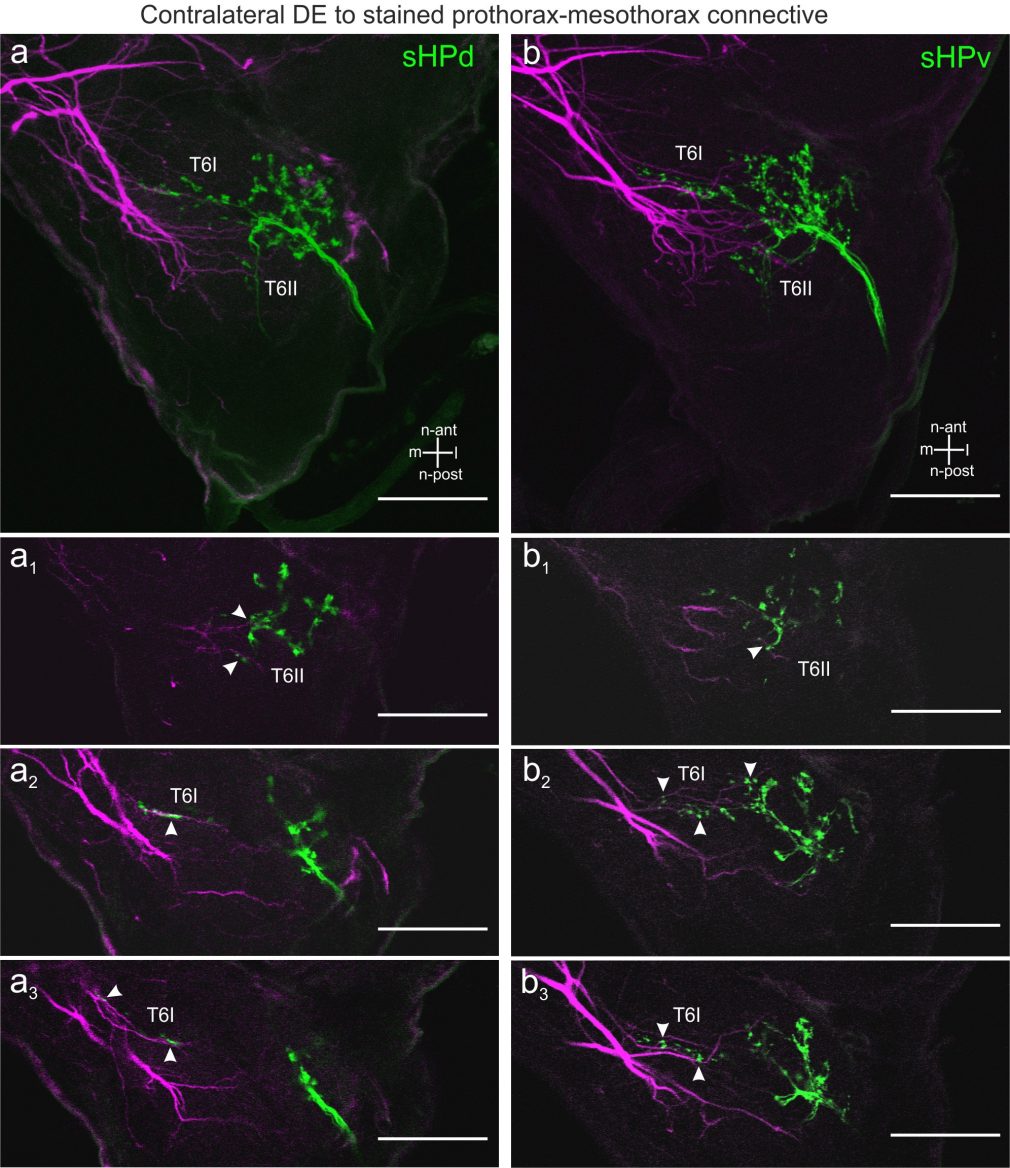
Potential contact sites between scapal HF afferents and DN branches in the DE of CRG, contralateral to the dyed prothorax-mesothorax connective. a: Longitudinal projection of sHPd afferents (green) and DN branches (magenta). a1-a3: Single images of (a) from n-dorsal to more n-ventral parts of the DL and tract T6I. For an explanation of the term neuraxis (n-) please see Fig 1. a1: Few fine DN branches enter the DL. Arrowheads indicate regions with overlap. Lower arrowhead marks vicinity of boutons in tract T6II with DN branches. a2-a3: DN branches entering the VFA (medial). Arrowheads label vicinity of sHPd afferents and DN branches in the medial-n-anterior tract 6I. b: Longitudinal projection of sHPv afferents (green) and DN branches (magenta). b1-b3: Single images of (b) from dorsal to more ventral parts of the DL. b1: Arrowheads mark DN branches in vicinity to sHPv afferents in tract T6II. b2-b3: Arrowheads indicate vicinity of DN branches with sHPv afferents in the anterior DL and along T6I. Scale bars = 100 μm.

In order to identify neuropil regions with potential contacts between hair field afferents (HF) and DNs, double stainings were made. Since the connective backfills (Figs 1–4) revealed DN branches close-by or invading the ipsi- and contralateral DL and VFA neuropils of the deutocerebrum (Fig 2C), we wondered whether central projections of antennal HFs overlap with branches of DNs. Indeed, double stainings such as the one shown in Fig 5 show DN branches contralateral to a dyed connective overlap with projections of scapal HF afferents (both with sHPd and sHPv). Vicinity of HF afferents and DN branches were found along the medial-n-anteriorly travelling HF afferents in tract T6I (Fig 5A2, 5A3, 5B2–5B3), in the medial-n-anterior DL (Fig 5B2) and in tract T6II (for description of HF afferent projections and terminology of tracts T6I and T6II see [33]). In tract T6II, afferents get close to DN branches immediately after they turn n-posteriorly to project towards the circumoesophageal connective (lower arrowheads; Fig 5A1 and 5B1).

**Fig 6:**
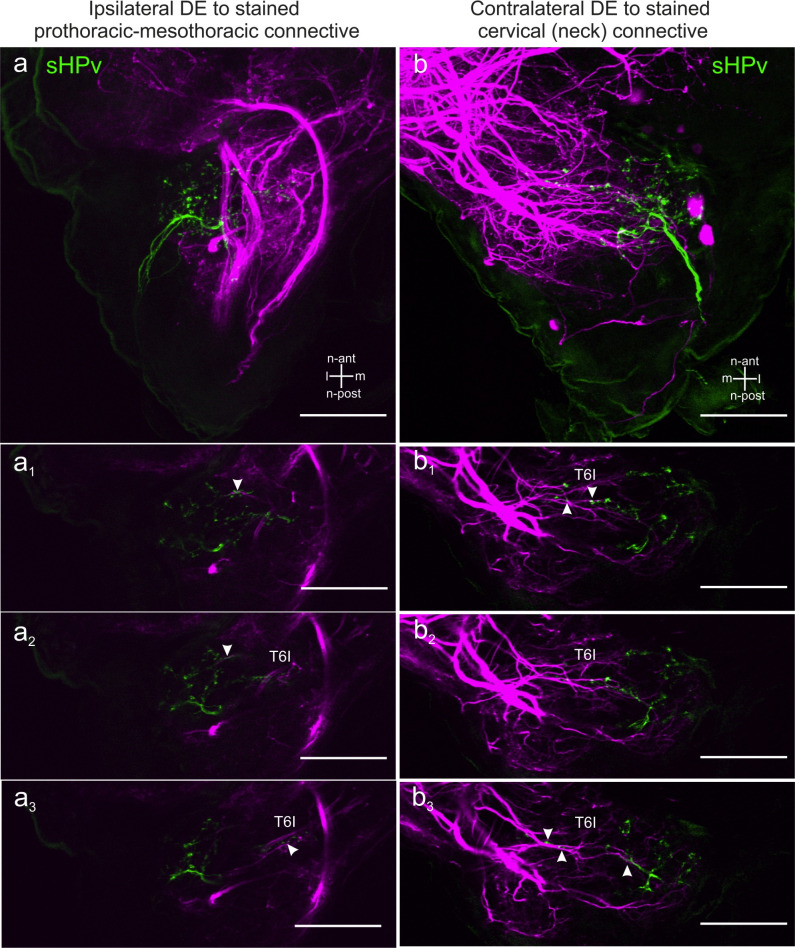
Potential contact sites between scapal HF afferents and of DN branches. a: CRG, ipsilateral to the dyed prothorax-mesothorax connective. Longitudinal projection of sHPv afferents (green) and DN branches (magenta). a1-a3: Single images of (a) from n-dorsal to more n-ventral parts of the DL. a1-a2: Few fine DN branches enter the DL. Arrowheads indicate possible overlap with HF afferent terminals. a3: Possible overlap of sHPv afferents and DN branches in the medial-n-anterior tract 6I (arrowheads). b: Deutocerebrum, contralaterally to a dyed neck connective. Longitudinal projection of sHPv afferents (green) and DN branches (magenta). b1-b3: Single images of (a), from n-dorsal to more n-ventral parts of the DL. For an explanation of the term neuraxis (n-) please see Fig 1. b1-b2: Arrowheads indicate DN branches in vicinity to sHPv afferents along T6I. b3: Arrowheads indicate vicinity of DNs with sHPv afferents in the anterior DL and along T6I. Scale bars = 100 μm.

Overall, there appear to be more contralateral than ipsilateral potential contact sites between HF afferents and DNs. In the ipsilateral DL, only one DN branch was found in the vicinity of sHPv afferents (Fig 6A1 and 6A2). Additional likely contact regions were identified along the medial-n-anteriorly travelling afferents in T6I (Fig 6A3).

An example showing the overlap of HF afferent with contralateral DN branches stained by backfilling a neck connective is shown in Fig 6. Numerous fine branches of DN branches invade the contralateral DL (Fig 6B1–6B3). In summary, DN branches were found in vicinity to HF afferents not only in tracts T6I and T6II, slightly outside the DL, fine branches of DNs or even ANs were found to project into the DL, too.

**Fig 7:**
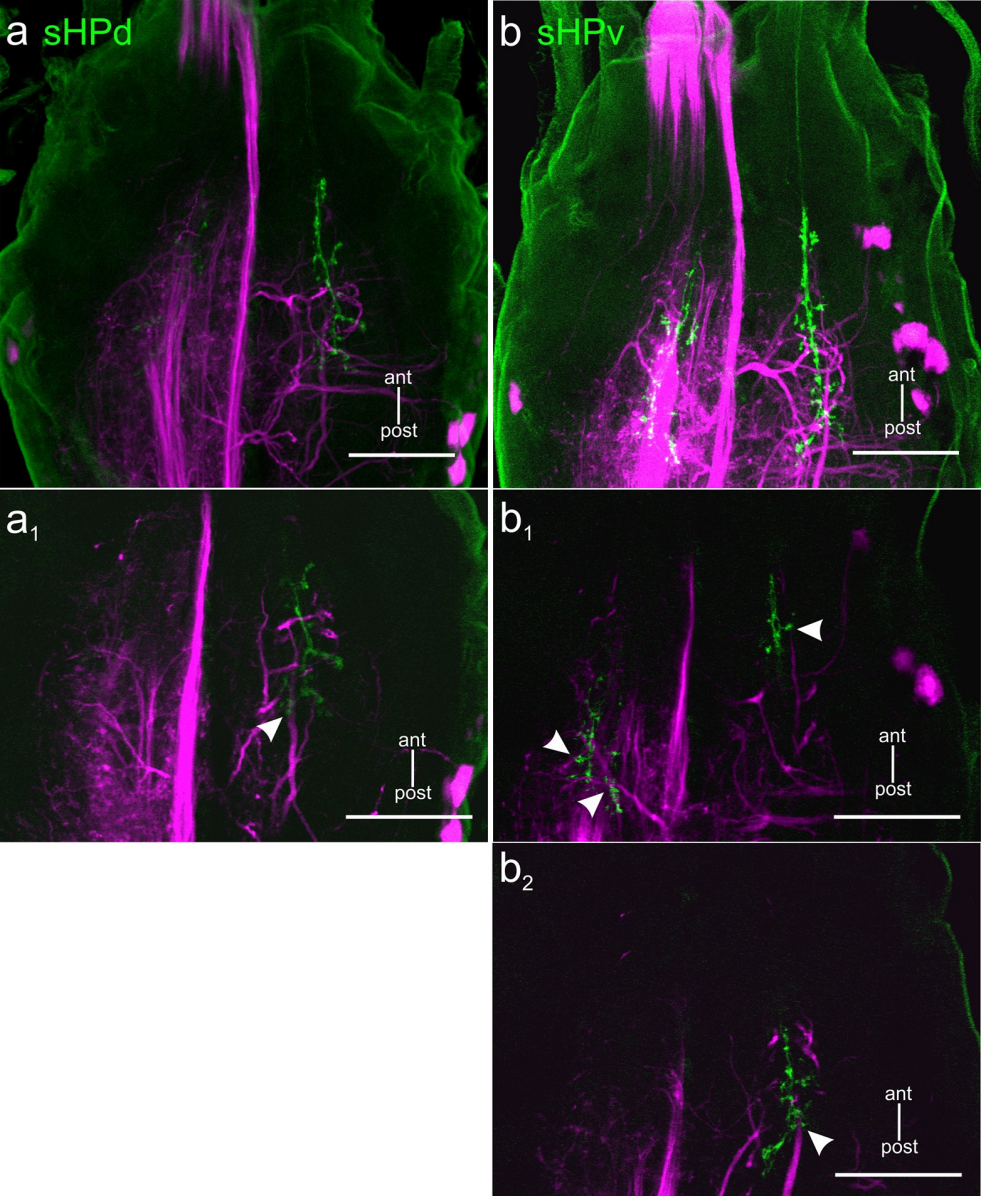
Potential contact sites between hair field afferents and DNs within the GNG. Double stainings of scapal HF afferents (green) and backfilled from the prothorax-mesothorax connective (magenta). a: Overview of GNG whole-mount with sHPd afferents (green) stained bilaterally (only the HF afferents on the contralateral side with respect to the stained connective were stained sufficiently). a1: Single image of a again reveals potential contact regions between afferents and DN branches near the terminal inverted-“V” structure of the afferents (arrowhead). Scale bars = 100 μm. b: Overview of another GNG whole-mount with sHPv afferents (green) stained bilaterally. b1-b2: Single images of (b), revealing regions where DN branches and HF afferents are in close vicinity, i.e., potential contact sites between them (arrowheads).

As shown by Goldammer and Dürr [33] (their Figs 1–4), antennal HF afferents not only project into the DE but also descend to the GNG. Our Fig 7 shows two specimens with stained DNs and HF afferents within the GNG, revealing further likely contact sites. Along the descending pathway of the antennal scapal HF fibres within the GNG, and particularly, close to the terminating inverted-“V” structure, terminal boutons of HF afferents can be found in the vicinity of branches of GNG DNs or intercalated interneurons. As a consequence, synaptic interaction sites between HF afferents and DNs may occur both in the CRG and in the GNG.

## Discussion

Backfills of the neck connectives revealed up to 205 pairs of DNs in the CRG (Fig 1) and up to 205 pairs of DNs in the GNG (Fig 3). Therefore, at least 410 pairs of neurons are involved in conveying information from the head ganglia to the prothoracic ganglion (Figs 1 and 2, Table 1). In the CRG, the stained cell bodies could be assigned to 19 soma clusters (eleven ipsilateral, seven contralateral, and one medial). The location of these clusters was described in relation to their neighbouring neuropils (Fig 2, Table 1).

Backfills of prothorax-mesothorax connectives revealed a much lower number of cell bodies, amounting to a total of 173 neuron pairs, 83 in the CRG and 90 in the GNG (Fig 4, Table 2). In the CRG, the cell bodies were assigned to 16 soma clusters (nine ipsilateral, six contralateral and one medial). Clusters i8, i9 and c7 were never found in these preparations, potentially because these clusters contain only DNs that terminate in the prothoracic ganglion. Overall, the number of DNs stained posterior to the prothoracic ganglion was less than half of the number stained at the neck connective. According to the numbers found, we estimate an upper bound of 60% of CRG DNs and 56% of GNG DNs to terminate in the prothoracic ganglion. This must be considered an upper bound because the longer dye travelling distance from the posterior connective cut as well as failures to fill DNs with thin axon diameter could result in a lower success rate of the stainings, leading to lower counts of stained DNs projecting to the mesothoracic ganglion.

Double stains of DNs and hair field afferents revealed overlapping branches both in the ipsilateral (Fig 6) and contralateral deutocerebrum (Figs 5 and 6) of the CRG. Moreover, our preparations confirm the finding of Ache et al. [26] in that there are overlapping branches in the mid part of the GNG (Fig 7). Since morphological apposition of afferent terminals and DN branches is a prerequisite for direct synaptic contacts between them, it is possible that two parallel pathways convey antennal afferent information to the prothoracic ganglion, potentially with as few as two synapses between antennal proprioceptors and thoracic motor networks: one via DNs of the CRG and another via DNs of the GNG. Recent ablation experiments have revealed the latter of these pathways to provide movement-related input from antennal hair field afferents to the DN cONv at the level of the gnathal ganglion [37].

### Descending interneurons (DNs) in stick insect CRG and GNG

Our finding of at least 205 CRG DNs is lower compared to 300 DNs reported for the blowfly brain [22], but in agreement for CRG DNs backfilled through neck connectives of the cricket, with at least 181 pairs of neurons [19], to the cockroach with at least 235 pairs [20]. Comparable CRG DN numbers, analysed via light microscopy, were reported for *Drosophila*, ranging from 172 pairs [24] to 206 pairs [23]. In contrast, only 106 DNs in the CRG and 88 DNs in the GNG were stained through neck connectives of cockroaches [21]. Numbers for stained GNG DNs in *Drosophila* range from 180 pairs [24] to 526 in total [23], whereas 153 GNG DNs were found in the locust [18]. However, since the latter study stained either neck connectives or prothorax-mesothorax connective, it is unclear if the numbers of GNG DNs counted were higher or lower than the numbers we found in stick insects.

Numbers of stained DNs projecting to the mesothoracic ganglia were not found in the literature. As a consequence, there is no staining data available for the fraction of DNs terminating in the prothoracic ganglion in other species. However, in the cockroach [45] backfilled the mesothorax-metathorax connective and found approximately 50 DN somata in the entire CRG and further 35 in the GNG, suggesting that 40 to 45 DN pairs project at least to the metathoracic ganglion. In conjunction with the 235 DN pairs found by the neck connective stains of cockroaches [20], this is reminiscent of the observed strong reduction of DNs that project beyond the prothorax in stick insects.

Nevertheless, the DN numbers of Tables 1 and 2 should be considered lower bounds for DNs conveying information from the brain to the thoracic ganglia. For instance, Okada et al. [20] estimated 284 CRG DNs by counting the maximum numbers of cell bodies of different clusters, whereas the total numbers per specimen ranged only between 160 and 212. Applying the same rationale to Tables 1 and 2 (see table rows labelled *max*.) also yields higher estimates than the maximum observed DN number per specimen: 238 pairs of CRG DNs (116%) projecting to the prothoracic ganglion and 94 pairs of CRG DNs (113%) projecting to the mesothoracic ganglion. Likely reasons for the variation in observed DN numbers in backfill studies are the long dye travelling distance, failure to fill thin axons, and counting errors in dense soma clusters.

Recently, a connectome dataset of a *Drosophila* male adult nerve chord [25] yielded a total of 1328 DNs. Approximately 125 pairs of these male *Drosophila* DNs are innervating all three thoracic leg neuropils, approximately 220 DN pairs are innervating multiple neuropils in all three thoracic neuromeres, and approximately 125 DN pairs are innervating the neck, wing, and haltere neuropils in the upper tectulum located in the first two thoracic neuromeres [25]. The innervation of the first thoracic neuromere is more complex than that of the other two, with approximately 50 pairs of additional DNs innervating the neck tectulum and approximately 60 pairs of additional DNs innervating the front leg neuropil [25]. In comparison, our reported numbers of 400 DN pairs in the stick insect CRG and GNG are much lower than in *Drosophila*.

### Soma clusters of DNs in the CRG

In general, the clustering of DN somata in stick insects is very similar to cell clusters in the cricket, cockroach, and *Drosophila*. Stick insect DNs were assigned to 19 soma clusters in the CRG. The locations of these clusters correspond to the 17 cluster locations described for the cricket (i1-i5, i5n, i6, i7a, i7b, i8, pi, and c1-c6; [19]) In contrast to the cricket, additional cell bodies were found in clusters i9 and c7. It is possible that neurons from cluster i9 in the stick insect resemble solitary neurons at a similar location described for the cricket [19]. When comparing our results with those for the cockroach, 15 out of 22 clusters of the cockroach [20] have matching locations in stick insects (i1-i5, i5n, i7a, i7b, i8, i9, pi, and c1-c4). Since Okada et al. [20] adjusted the nomenclature of Staudacher [19] for the cockroach, newly identified or renamed clusters in the cockroach appear to be homologues of stick insect clusters. These cockroach clusters are c7 (c5 in the stick insect), c8 (c7 in the stick insect), and c9 (c6 in the stick insect). Additionally, Okada et al. [20] described two clusters (i6a and c6) near the β-lobes of the mushroom bodies. Somata at a similar location were also found in stick insects, but only very rarely (see question mark and unnamed soma clusters in Figs 1 and 2). However, the locations of specific soma clusters can vary slightly. For instance, cluster i1 in stick insects is located laterally and more n-posteriorly in the PR than those of cockroaches and crickets, where it can be found near the ipsilateral calyx of the mushroom body. For *Drosophila*, cell clusters were reported on corresponding locations described for the cricket and cockroach (i1-4, c1-c4, i5, i5n, i7a and b, c7, and pi; [23]), which can be found in the stick insect, too. However, an additional cluster in *Drosophila*, between the anterior ventrolateral PR and the antennal lobe, could correspond to cluster i6 in the stick insect.

### Distribution of arborisations in the CRG

Stainings of connectives revealed distinct regions in the stick insect brain with extensive dendritic arborisations. Whether these arborisations belong to primary ascending sensory neurons (e.g. [46]), ANs, or DNs is not discernible. The most extensive dendritic arborisations were observed in the ipsilateral posterior lateral protocerebrum (PLP), the ipsilateral posterior ventrolateral protocerebrum (PVLP), and in more medial parts of the n-posterior protocerebrum (PR) of both CRG hemispheres (Figs 1 and 2). The medial PR regions appear similar to the inferior neuropils (INP) described for *Drosophila* [27]. Dendritic branches were also found in the ipsilateral lateral accessory lobe (LAL), ipsilateral posterior slope (PS) and in the VFA and DL neuropils of both CRG hemispheres (see below). The PS is a very important neuropil for descending signals in the silk moth [47] and *Drosophila* [24], too. The lateral and medial PR neuropils receive signals from mushroom body output neurons [48]. Similar to our data on stick insects, Okada et al. [20] observed numerous dendritic branches in the PR of the cockroach. They suggested that at least some mushroom body output neurons have direct connections to DNs. The stick insect PLP and PVLP neuropils are innervated by two thick fibre bundles (e.g., Fig 2). Therefore, it is likely that a considerable overlap of AN with DN branches occurs within these regions. Similarly, Okada et al. [20] reported an overlap of AN and DN arborisations in the PR of cockroaches. They suggested that this overlap may indicate the existence of a rapid feedback transmission from ANs to DNs, which could be important to shape descending signals for motor control. The LAL is thought to play a role in visual integration and it appears to be a neuropil which connects ascending and descending pathways with the central complex, the anterior optic tubercles, and posterior parts of the PR [43]. Lesioning experiments of the LAL in cockroaches resulted in striking abnormalities in obstacle negotiation and turning behaviour, suggesting an important output function for descending motor commands [49]. In the stick insect, we observed no dendritic arborisations in the AL, the central complex, or the mushroom bodies. This is in agreement with studies on crickets [19], cockroaches [20], and *Drosophila* [23, 24].

### Functional aspects of DNs in insects

Our results show that DN branches and antennal HF afferent terminals arborise in two regions: Within the CRG this is the DL of the deutocerebrum (Figs 5 and 6), within the GNG this is the mid part (Fig 7). Given the high degree of overlap among the afferent terminal regions of all seven antennal hair fields [33], this suggests that postural information of the antenna could be transmitted to thoracic ganglia by two parallel pathways: one involving synaptic contacts with CRG DNs in the DL, and another involving GNG DNs. Concerning the DL pathway, Staudacher and Schildberger [50] reported that branches of the cricket DN DBNi2-1 overlap with antennal afferent terminals in the DL. This is similar to the situation shown in Fig 6A–6A3, where a DN branch and HF afferents ramify in vicinity, ipsilaterally to the stained connective. Concerning the GNG pathway, our results corroborate findings by [26], that both an ipsilaterally (iONv) and a contralaterally (cONv) descending neuron have arborisations in close vicinity to afferent terminals of antennal hair fields in the GNG. Owing to the short delay of stimulus-induced spikes in iONv and cONv, Ache et al. [26] suggested that indeed those two DNs were part of a direct antenno-mechanosensory pathway to the prothoracic ganglion. Nevertheless, it is important to note that the neuroanatomical observations presented in Figs 5–7 need to be considered with care due to the limitations in confocal microscopy [51]. Our results are not sufficient to prove the existence of direct functional synaptic contacts, but delimit the regions in the CRG and GNG where such contacts are likely. Therefore, further anatomical investigations are necessary for evidence of synaptic contacts.

Until today, few brain DNs have been identified in different insect species that receive mechanosensory signals from antennal sense organs. An identified giant descending neuron (GDN) in Dipterans receives inputs from visual neurons and mechanosensory inputs from antennal sense organs [52]. In the cricket and cockroach, the descending brain neurons with a contralateral descending axon and branches in the DE respond to antennal touch with short latencies (cricket: DBNc1-2 and DBNc2-2 [53]; cockroach: DMIa-1 [45, 54]). According to their soma positions these neurons are likely to be found in cluster c1 and c3 in the stick insect (Figs 1A and 3A). In contrast to the contralateral descending neurons in crickets, DN branches in stick insects were observed not only in the VFA but also near or within the DL and along T6I and T6II, where an overlap was found with antennal HF afferents (Figs 5 and 6B). The extensive branching of DN branches in the stick insect DL and VFA neuropils could indicate that these neurons receive several signals from a variety of different mechanoreceptors distributed along all antennal segments. Contralaterally descending CRG DNs of crickets and cockroaches have been proposed to have a function in the neural control of locomotion. The cockroach DMIs are assumed to be involved in tactually elicited escape turns [45]. The cricket DBNs were suggested to evoke fast turning responses towards or away from obstacles, predators or conspecifics [53]. Intracellular recordings of DBNi2-1 revealed that the spike frequency correlated with walking velocity and steering towards the contralateral side [55]. A discovered population of DNs in *Drosophila* triggers the initiation of backward directed walking (MDM neurons [56, 57]). According to their soma positions in the *Drosophila* brain these neurons can possibly be found in the pi cluster of stick insects.

Ipsilaterally descending neurons of crickets (DBNi1-2 and DBNi2-1) respond to deflection of the pedicel or the flagellum of the antenna [53, 58]. Neurons with similar locations and basic structure can possibly be found in clusters c1 and c2 in the stick insect CRG (Figs 1A and 3A). In contrast to stick insect DNs, the cricket DBNs have fan-shaped branches mostly in the ventral posterior DE region, where the VFA is located [53, 59]. The exception is the ipsilaterally descending neuron DBNi2-1 that has branches in the DE more dorsally than the other DBNs [53, 59]. Another DN of crickets was reported to respond to moving gratings in the visual field, whereas the neuronal activity of another descending neuron was correlated with the translational velocity of the walk of the animal [60].

Concerning GNG DNs, two neurons were identified in the cricket (SOG-dc1 and SOG-dc2 [53], and one in the cockroach (DMIb-1 [45]). All three of these project to all thoracic ganglia and respond to mechanical stimulation of the antenna with an increase in activity. Although their arborisation patterns within the GNG have some similarity with that found for stick insect neuron cONv [26], the differences in experimental stimulation protocols do not allow a detailed comparison of their tuning to exteroceptive and proprioceptive mechanosensory cues. The stick insect neuron OFFv has a high resting activity and responds to antennal stimulation with a decrease in activity [26]. For comparison with the soma locations shown in Figs 3 and 4C, the cockroach neuron DMIb-1 and the stick insect neurons cONv and OFFv have their soma in the contralateral posterior third of the GNG, the cricket neurons SOG-dc1 and SOG-dc2 in the contralateral medial third of the GNG, and the stick insect iONv [26] in the ipsilateral medial third of the GNG.

In summary, our results show that at least 400 pairs of descending interneurons connect the head ganglia to the thoracic ganglia, with approximately half of this population having their soma in the CRG. Furthermore, we show that a substantial fraction of DNs do not project beyond the prothoracic ganglion (with 56 to 60% being an upper bound to this estimate), suggesting much stronger neural input to the motor networks of the prothoracic ganglion than to the other two thoracic ganglia. Finally, our results show that synaptic interactions between antennal proprioceptive afferents and DNs could likely occur in the DL of the deutocerebrum, and corroborate findings by [26] regarding potential synaptic contacts in the GNG. Given the behavioural relevance of the antennal tactile sense for adaptive locomotion in nocturnal, canopy-dwelling, obligatory walking stick insects (they can neither jump nor fly), and in conjunction with electrophysiological characterisation [34, 26], ablation studies [37] and computational modelling [61] of stick insect DNs, our neuroanatomical data support the view of a fast cephalothoracic pathway conveying antennal postural information. This could be part of the anatomical substrate underlying fast, tactually elicited, aimed reach-to-grasp movements of the front leg in climbing stick insects [7].

## Supporting information

S1 Fig Additional depth colour-coded specimen with CRG DNs stained through a neck connective. Depth encoded from n-dorsal to n-ventral as blue to red. For an explanation of the term neuraxis (n-) please see Fig 1. Scale bar = 100 μm.
(TIF)

## References

[1] Photohorotaxis Kalmus H., eine neue Reaktionsart, gefunden an den Eilarven von *Dixippus morosus*. Z vergl Physiol. 1937; 24, pp. 644–655. doi: 10.1007/BF00592302

[2] Jander R, Volk-Heinrichs I. Das Strauch-spezifische Perceptor-System der Stabheuschrecke (*Carausius morosus*). Z vergl Physiol. 1970; 70, pp. 425–447. doi: 10.1007/bf00298197

[3] Frantsevich I, Frantsevich L. Space constancy in form perception by the stick insect. Naturwiss. 1996; 83 (7), pp. 323–324. doi: 10.1007/BF01152214

[4] Rosano H, Webb B. A dynamic model of thoracic differentiation for the control of turning in the stick insect. Biol Cybern. 2007; 97 (3), pp. 229–246. doi: 10.1007/s00422-007-0170-4 17647010

[5] Dürr V, Ebeling W. The behavioural transition from straight to curve walking: kinetics of leg movement parameters and the initiation of turning. J Exp Biol. 2005; 208 (12), pp. 2237–2252. doi: 10.1242/jeb.01637 15939767

[6] Gruhn M, Zehl L, Büschges A. Straight walking and turning on a slippery surface. J Exp Biol. 2009; 212, pp. 194–209. doi: 10.1242/jeb.018317 19112138

[7] Schütz C, Dürr V. Active tactile exploration for adaptive locomotion in the stick insect. Phil Trans R Soc Lond B. 2011; 366 (1581), pp. 2996–3005. doi: 10.1098/rstb.2011.0126 21969681 PMC3172591

[8] Krause AF, Dürr V. Active tactile sampling by an insect in a step-climbing paradigm. Front Behav Neurosci. 2012; 6 (30), pp. 1–17. doi: 10.3389/fnbeh.2012.00030 22754513 PMC3384986

[9] Gruhn M, Rosenbaum P, Bockemühl T, Büschges A. Body side-specific control of motor activity during turning in a walking animal. *eLife*. 2016; 5:e13799. doi: 10.7554/eLife.13799 27130731 PMC4894755

[10] Hammel E, Mantiaris C, Schmitz J, Büschges A, Gruhn M. Thorax-Segment- and leg-segment-specific motor control for adaptive behavior. Front Physiol. 2022; 13:883858. doi: 10.3389/fphys.2022.883858 35600292 PMC9114818

[11] de Sinéty R. Prétendue absorption de graisse par le jabot chez les Blattes (Orth.) Bull Soc Ent Fr. 1901; 1901:255–6.

[12] Wendler G. Laufen und Stehen der Stabheuschrecke: Sinnesborsten in den Beingelenken als Glieder von Regelkreisen. Z vergl Physiol. 1964; 48, pp. 198–250. doi: 10.1007/BF00297860

[13] Bässler U. Neural basis of elementary behavior in stick insects. Berlin Heidelberg New York: Springer (Studies in Brain Function), 1983.

[14] Pattern Graham D. and control of walking in insects. Adv Insect Physiol. 1985; 18, pp. 31–140. doi: 10.1016/S0065-2806(08)60039-9

[15] Büschges A, Gruhn M. Mechanosensory feedback in walking: From joint control to locomotor patterns. Adv Insect Physiol. 2007; 34, pp. 193–230. doi: 10.1016/S0065-2806(07)34004-6

[16] Dürr V, Theunissen LM, Dallmann CJ, Hoinville T, Schmitz J. Motor flexibility in insects: Adaptive coordination of limbs in locomotion and near-range exploration. Behav Ecol Sociobiol. 2018; 72 (1), p. 15. doi: 10.1007/s00265-017-2412-3

[17] Büschges A. Lessons for circuit function from large insects: towards understanding the neural basis of motor flexibility. Curr Opin Neurobiol. 2012; 22 (4), pp. 602–608. doi: 10.1016/j.conb.2012.02.003 22386530

[18] Kien J, Fletcher WA, Altman JS, Ramirez JM, Roth U. Organization of Intersegmental Interneurons in the subesophageal ganglion of *Schistocerca gregaria* (Forksal) and *Locusta migratoria migratorioides* (Reiche and Fairmaire) (Acrididae, Orthoptera. Int J Insect Morphol Embryol. 1990; 19 (1), pp. 35–60. doi: 10.1016/0020-7322(90)90029-O

[19] Staudacher E. Distribution and morphology of descending brain neurons in the cricket *Gryllus bimaculatus*. Cell Tissue Res. 1998; 294 (1), pp. 187–202. doi: 10.1007/s004410051169 9724469

[20] Okada R, Sakura M, Mizunami M. Distribution of dendrites of descending neurons and its implications for the basic organisation of the cockroach brain. J Comp Neurol. 2003; 458 (2), pp. 158–174. doi: 10.1002/cne.10580 12596256

[21] Gal R, Libersat F. New vistas on the initiation and maintenance of insect motor behaviors revealed by specific lesions of the head ganglia. J Comp Physiol A. 2006; 192 (9), pp. 1003–1020. doi: 10.1007/s00359-006-0135-4 16733727

[22] Strausfeld NJ, Lee JK. Neuronal basis for parallel visual processing in the fly. Vis Neurosci. 1991; 7 (1–2), pp. 13–33. doi: 10.1017/s0952523800010919 1931797

[23] Hsu CT, Bhandawat V. Organization of descending neurons in *Drosophila* melanogaster. Scientific Report. 2016; 6 (1), p. 20259. doi: 10.1038/srep20259 26837716 PMC4738306

[24] Namiki S, Dickinson MH, Wong AM, Korff W, Card GM. The functional organization of descending sensory-motor pathways in *Drosophila*. eLife. 2018; 7, e34272. doi: 10.7554/eLife.34272 29943730 PMC6019073

[25] Cheong HSJ, Eichler K, Stuerner T, Asinof SK, Champion AS, Marin EC, et al. Transforming descending input into behavior: The organization of premotor circuits in the *Drosophila* Male Adult Nerve Cord connectome. bioRxiv 2023.06.07.543976; doi: 10.1101/2023.06.07.543976

[26] Ache JM, Haupt SS, Dürr V. A direct descending pathway informing locomotor networks about tactile sensor movement. J Neurosci. 2015; 35 (9), pp. 4081–4091. doi: 10.1523/JNEUROSCI.3350-14.2015 25740535 PMC6605569

[27] Ito K, Shinomiya K, Ito M, Armstrong JD, Boyan G, Hartenstein V, et al. A systematic nomenclature for the insect brain. Neuron. 2014; 81 (4), pp. 755–765. doi: 10.1016/j.neuron.2013.12.017 24559671

[28] Theunissen LM, Dürr V. Insects use two distinct classes of steps during unrestrained locomotion. PLOS one. 2013; 8 (12), e85321. doi: 10.1371/journal.pone.0085321 24376877 PMC3871641

[29] Grabowska M, Godlewska E, Schmidt J, Daun-Gruhn S. Quadrupedal gaits in hexapod animals—inter-leg coordination in free-walking adult stick insects. J Exp Biol. 2012; 215 (24), pp. 4255–4266. doi: 10.1242/jeb.073643 22972892

[30] Theunissen LM, Vikram S, Dürr V. Spatial co-ordination of foot contacts in unrestrained climbing insects. J Exp Biol. 2014; 217 (18), pp. 3242–3253. doi: 10.1242/jeb.108167 25013102

[31] Dürr V. Stereotypic leg searching-movements in the stick insect: Kinematic analysis, behavioural context and simulation. J Exp Biol. 2001; 204 (9), pp. 1589–1604. doi: 10.1242/jeb.204.9.1589 11398748

[32] Berg EM, Büschges A, Schmidt J. Single perturbations cause sustained changes in searching behavior in stick insects. J Exp Biol. 2013; 216 (6), pp. 1064–1074. doi: 10.1242/jeb.076406 23197090

[33] Goldammer J, Dürr V. Proprioceptive input to a descending pathway conveying antennal postural information: Terminal organisation of antennal hair field afferents. Arthropod Struct Dev. 2018; 47, pp. 465–481. doi: 10.1016/j.asd.2018.07.001 30076912

[34] Ache JM, Dürr V. Encoding of near-range spatial information by descending interneurons in the stick insect antennal mechanosensory pathway. J Neurophysiol. 2013; 110 (9), pp. 2099–2112. doi: 10.1152/jn.00281.2013 23926042

[35] Okada J, Toh Y. Peripheral representation of antennal orientation by the scapal hair plate of the cockroach *Periplaneta americana*. J Exp Biol. 2001; 204 (24), pp. 4301–4309. doi: 10.1242/jeb.204.24.4301 11815654

[36] Krause AF, Winkler A, Dürr V. Central drive and proprioceptive control of antennal movements in the walking stick insect. J Physiol Paris. 2013; 107 (1–2), pp. 116–129. doi: 10.1016/j.jphysparis.2012.06.001 22728470

[37] Jaske B, Lepreux G, Dürr V. Input of hair field afferents to a descending interneuron. J Neurophysiol. 2021; 126 (2), pp. 398–412. doi: 10.1152/jn.00169.2021 34161139

[38] Goldammer J, Büschges A, Schmidt J. Motoneurons, DUM cells, and sensory neurons in an insect thoracic ganglion: A tracing study in the stick insect *Carausius morosus*. J Comp Neurol. 2012; 520 (2), pp. 230–257. doi: 10.1002/cne.22676 21618233

[39] Klagges BRE, Heimbeck G, Godenschwege TA, Hofbauer A, Pflugfelder GO, Reifegerste R, et al. Invertebrate synapsins: A single gene codes for several isoforms in *Drosophila*. J Neurosci. 1996; 16 (10), pp. 3154–3165. doi: 10.1523/JNEUROSCI.16-10-03154.1996 8627354 PMC6579133

[40] Suzuki H. Antennal movements induced by odour and central projection of the antennal neurones in the honey-bee. J Insect Physiol. 1975; 21, pp. 831–847. doi: 10.1016/0022-1910(75)90012-8

[41] Dürr V, König Y, Kittmann R. The antennal motor system of the stick insect *Carausius morosus*: anatomy and antennal movement pattern during walking. J Comp Physiol A. 2001; 187 (2), pp. 131–144. doi: 10.1007/s003590100183 15524001

[42] Homberg U, Montague RA, Hildebrand JG. Anatomy of antenno-cerebral pathways in the brain of the sphinx moth *Manduca sexta*. Cell Tissue Res. 1988; 254 (2), pp. 255–281. doi: 10.1007/BF00225800 3197087

[43] Homberg U. Flight-correlated activity changes in neurons of the lateral accessory lobes in the brain of the locust *Schistocerca gregaria*. J Comp Physiol A. 1994; 175 (5), pp. 597–610. doi: 10.1007/BF00199481

[44] Stolz T, Diesner M, Neupert S, Hess ME, Delgado-Betancourt E, Pflüger HJ, et al. Descending octopaminergic neurons modulate sensory-evoked activity of thoracic motor neurons in stick insects. J Neurophysiol. 2019; 122: 2388–2413. doi: 10.1152/jn.00196.2019 31619113

[45] Burdohan JA, Comer CM. Cellular organization of an antennal mechanosensory pathway in the cockroach, *Periplaneta americana*. J Neurosci. 1996; 16 (18), pp. 5830–5843. doi: 10.1523/JNEUROSCI.16-18-05830.1996 8795635 PMC6578969

[46] Tsubouchi A, Yano T, Yokoyama TK, Murtin C, Otsuna H, Ito K. Topological and modality-specific representation of somatosensory information in the fly brain. Science. 2017; Nov 3;358(6363):615–623. doi: 10.1126/science.aan4428 29097543

[47] Namiki S, Wada S, Kanzaki R. Descending neurons from the lateral accessory lobe and posterior slope in the brain of the silkmoth Bombyx mori. Scientific Reports. 2018; 8 (1), p. 9663. doi: 10.1038/s41598-018-27954-5 29941958 PMC6018430

[48] Li YS, Strausfeld NJ. Multimodal efferent and recurrent neurons in the medial lobes of cockroach mushroom bodies. J Comp Neurol. 1999; 409 (4), pp. 647–663. doi: 10.1002/(sici)1096-9861(19990712)409:4&lt;647::aid-cne9&gt;3.0.co;2-3 10376745

[49] Harley CM; Ritzmann RE. Electrolytic lesions within central complex neuropils of the cockroach brain affect negotiation of barriers. J Exp Biol. 2010; 213 (16), pp. 2851–2864. doi: 10.1242/jeb.042499 20675555

[50] Staudacher E; Schildberger K. Gating of sensory responses of descending brain neurons during walking in crickets. J Exp Biol. 1998; 201, pp. 559–572. doi: 10.1242/jeb.201.4.559 9438831

[51] Pawley JB. (Ed.) Handbook of biological confocal microscopy. New York: Springer. 2006.

[52] Bacon JP, Strausfeld NJ. The dipteran Giant fibre pathway: neurons and signals. J Comp Physiol. 1986; 158 (4), pp. 529–548.

[53] Schöneich S, Schildberger K, Stevenson PA. Neuronal organization of a fast-mediating cephalothoracic pathway for antennal-tactile information in the cricket (*Gryllus bimaculatus* DeGeer). J Comp Neurol. 2011; 519, pp. 1677–1690. doi: 10.1002/cne.22594 21452239

[54] Burdohan JA, Comer CM. An antennal-derived mechanosensory pathway in the cockroach: descending interneurons as a substrate of evasive behaviour. Brain Res. 1990; 535 (2), pp. 347–352. doi: 10.1016/0006-8993(90)91623-O 2073615

[55] Zorovic M, Hedwig B. Descending brain neurons in the cricket *Gryllus bimaculatus* (de Geer): auditory responses and impact on walking. J Comp Physiol A. 2013; 199 (1), pp. 25–34. doi: 10.1007/s00359-012-0765-7 23104703

[56] Bidaye SS, Machacek C, Wu Y, Dickson BJ. Neuronal control of *Drosophila* walking direction. Science. 2014; 344, pp. 97–101. doi: 10.1126/science.1249964 24700860

[57] Feng K, Sen R, Minegishi R, Dübbert M, Bockemühl T, Büschges A, et al. Distributed control of motor circuits for backward walking in Drosophila. Nat Commun. 2020; Dec 2;11(1):6166. doi: 10.1038/s41467-020-19936-x 33268800 PMC7710706

[58] Gebhardt MJ, Honegger HW. Physiological characterisation of antennal mechanosensory descending interneuons in an insect (*Gryllus bimaculatus*, *Gryllus campestris*) brain. J Exp Biol. 2001; 204 (13), pp. 2265–2275. doi: 10.1242/jeb.204.13.2265 11507110

[59] Staudacher E, Schildberger K. A newly described neuropile in the deuterocerebrum of the cricket: Antennal afferents and descending interneurons. Zoology: Analysis of Complex Systems. 1999; 102 (2), pp. 212–226.

[60] Böhm H, Schildberger K. Brain neurons involved in the control of walking in the cricket *Gryllus bimaculatus*. J Exp Biol. 1992; 166, pp. 113–130. doi: 10.1242/jeb.166.1.113

[61] Ache JM, Dürr V. A computational model of a mechanoreceptive descending pathway involved in active tactile sensing. PLoS Comput Biol. 2015; 11 (7), e1004263–pp. 1–27. doi: 10.1371/journal.pcbi.1004263 26158851 PMC4497639

