# Supplementary figures and images for: Descending interneurons of the stick insect connecting brain neuropiles with the prothoracic ganglion
Source: PLoS One. 2023 Aug 31;18(8):e0290359. doi: 10.1371/journal.pone.0290359 (PMC10470933; doi:10.1371/journal.pone.0290359)

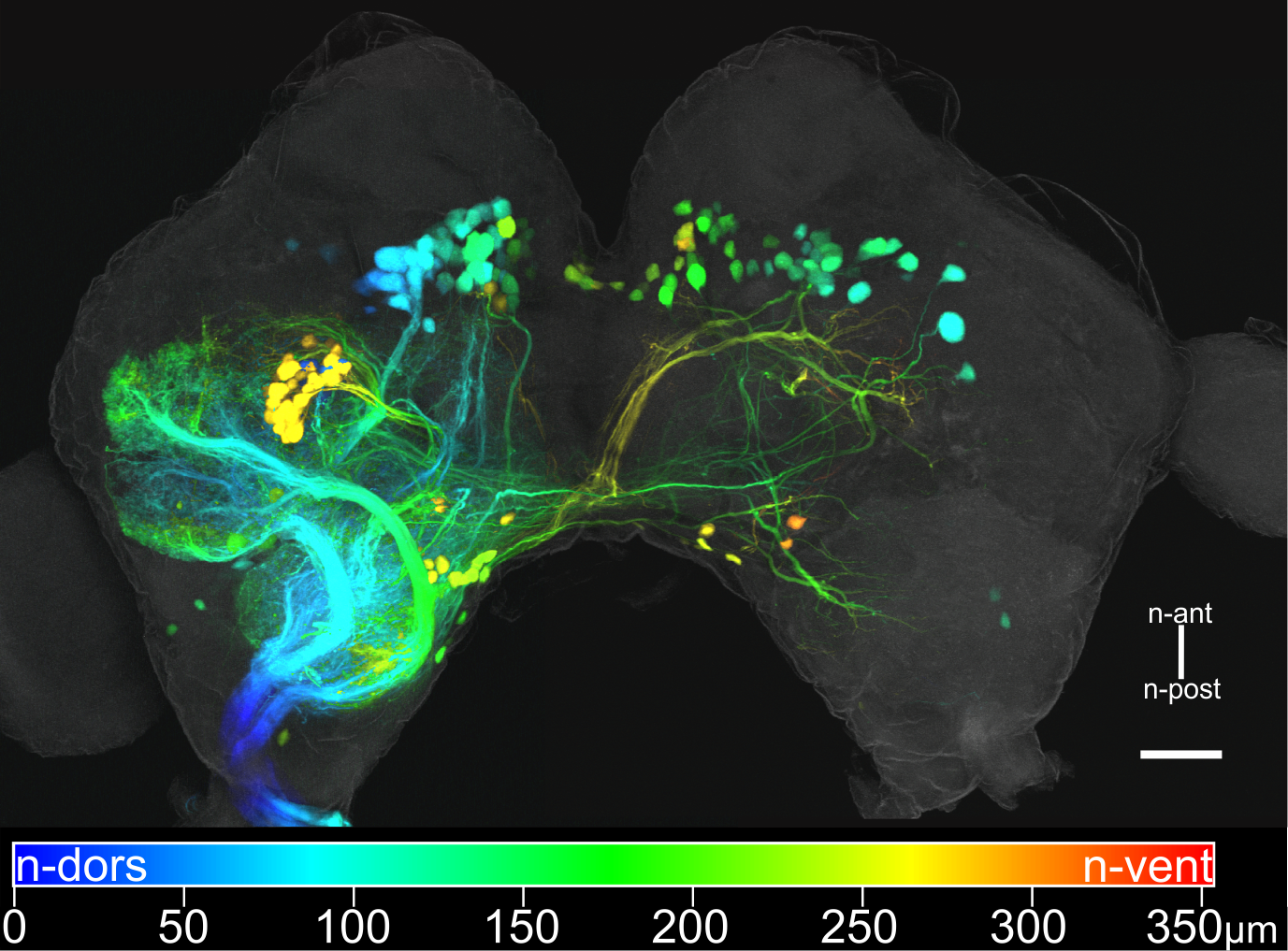

Supplement: S1 Fig — Depth encoded from n-dorsal to n-ventral as blue to red. For an explanation of the term neuraxis (n-) please see Fig 1. Scale bar = 100 μm. (TIF) [file pone.0290359.s001.tif]
